# Successful hemostasis of bleeding gastric inflammatory fibroid polyp by endoscopic treatment in a patient with severe COVID-19

**DOI:** 10.1007/s12328-021-01402-w

**Published:** 2021-04-11

**Authors:** Ayako Murota, Shinji Yoshi, Ryu Okuda, Sae Oowada, Tsukasa Yamakawa, Tomoe Kazama, Daisuke Hirayama, Keisuke Ishigami, Hiro-O. Yamano, Eichi Narimatu, Shintaro Sugita, Tadashi Hasegawa, Hiroshi Nakase

**Affiliations:** 1 Department of Gastroenterology and Hepatology, Sapporo Medical University School of Medicine, S-1, W-16, Chuo-ku, Sapporo, 063-8543 Japan; 2 Sapporo Medical University School of Medicine, Sapporo, Japan; 3 Department of Emergency Medicine, Sapporo Medical University School of Medicine, Sapporo, Japan; 4 Department of Surgical Pathology, Sapporo Medical University School of Medicine, Sapporo, Japan

**Keywords:** COVID-19, Inflammatory fibroid polyp, Hemostasis

## Abstract

The coronavirus disease-2019 (COVID-19) has rapidly become a pandemic, resulting in a global suspension of non-emergency medical procedures such as screening endoscopic examinations. There have been several reports of COVID-19 patients presenting with gastrointestinal symptoms such as diarrhea and vomiting. In this report, we present a case of successful hemostasis of bleeding gastric inflammatory fibroid polyp by endoscopic treatment in a patient with severe COVID-19. The case was under mechanical ventilation with extracorporeal membrane oxygenation (ECMO), and the airway was on a closed circuit. This indicates that COVID-19 is associated with not only lung injury but also intestinal damage, and that proper protective protocols are essential in guaranteeing the best outcomes for patients and clinical professionals during this pandemic.

## Introduction

At the end of 2019, severe acute respiratory syndrome coronavirus 2 (SARS-CoV-2) was identified as the cause of a cluster of pneumonia cases in the city of Wuhan, China; since this initial report, SARS-CoV-2 has rapidly spread worldwide due to its high infectivity. The disease caused by this novel virus was named coronavirus disease 2019 (COVID-19). COVID-19 primarily manifests as a lung infection with symptoms ranging from mild respiratory infection to severe pneumonia; typically presenting with fever, dyspnea, cough, myalgia, and fatigue [1, 2]. However, some patients with COVID-19 have presented with gastrointestinal (GI) symptoms such as diarrhea, nausea, vomiting, and abdominal pain. Additionally, COVID-19 presents as isolated GI symptoms before developing respiratory infection symptoms because diarrhea can precede other symptoms by a few days. A recent report demonstrates that GI bleeding was present in approximately 4–13.7% of COVID-19 cases [3]. However, there has been no report regarding bleeding from a gastric polyp in a patient with COVID-19.

Herein, we present a severe COVID-19 patient under mechanical ventilation with extracorporeal membrane oxygenation (ECMO) with GI bleeding from a gastric inflammatory fibroid polyp and successfully performed hemostasis by the endoscopic resection.

## Case presentation

A 68-year-old man presented to a local hospital because of cough and low-grade fever over the previous five days. He had a history of hypertension and diabetes mellitus. A nasopharyngeal swab specimen was collected and reported positive for COVID-19. Three days later, he was hospitalized after developing severe respiratory fatigue which required intubation and mechanical ventilation. Despite optimal ventilation management, by day 5 post hospitalization, his respiratory symptoms worsened and resulted in hypoxemic respiratory failure, with blood oxygen saturation of 91% on mechanical ventilation. Thus, he was transferred to Sapporo Medical University Intensive Care Unit and started on venovenous extracorporeal membrane oxygenation (VV-ECMO). His vital signs were as follows: body temperature of 37.5 °C, blood pressure of 125/82, pulse rate of 95 beats/min, respiratory rate of 12 breaths/min, oxygen saturation of 91% on mechanical ventilation. His laboratory data showed that the coagulation test was normal and platelet count was 251,000/µl (Table 1).

**Table 1.** Laboratory test results of the patients

		The day of admission	The day of EMR	The day after EMR
WBC	µL	10,000	12,900	11,400
RBC	×10^6^/µL	3.74	3.87	4.04
Hb	g/dL	9.3	10.1	10.9
Ht	%	28.9	31.2	32
MCV	fL	77.3	80.6	79.2
MCH	pg	24.9	26.1	27
MCHC	g/dL	32.2	32.4	34.1
Plt	µL	251,000	125,000	98,000
CRP	mg/dL	37.92	18.64	17.41
TP	g/dL	6.8	5.3	4.9
Alb	g/dl	1.9	1.8	1.8
AST	U/L	46	54	88
ALT	U/L	22	25	44
BUN	mg/dL	50	24	26
CRE	mg/dL	1.45	1.05	0.99
Fe	µg/dL	40	-	-
TIBC	µg/dL	168	-	-
Ferritin	ng/mL	568	640	-
PT	%	87.6	92.4	95.8
PTINR		1.06	1.04	1.01
APTT	sec	27.5	29.2	31.5
FBG	mg/dL	>800	401	368
FDP	µg/mL	23.2	7.5	8.6
DD	µg/mL	16.6	4.7	5.4
ATIII	%	54	77	79
*Blood gas analysis*				
pH		7.398	7.397	7.436
pO_2_	mmHg	63.0	47.3	42.8
pCO_2_	mmHg	39.6	49.0	45.0
ABE	mmol/L	−0.3	4.4	5.3
SBE	mmol/L	−0.3	4.8	5.6
Lac	mmol/L	2.2	1.3	1.2

*EMR* endoscopic mucosal resection; *WBC* white blood cell; *RBC* red blood cell; *Hb* hemoglobin; *Ht* hematocrit; *MCV* mean corpuscular volume; *MCH* mean corpuscular hemoglobin; *MCHC* mean corpuscular hemoglobin concentration; *Plt* platelet; *CRP* C-reactive protein; *TP* total protein; *AST* alanine aminotransferase; *ALT* aspartate aminotransferase; *BUN* blood uric nitrogen; *CRE* creatinine; *Fe* Ferrum; *TIBC* total iron binding capacity; *PT* prothrombin time; *APTT* activated partial thromboplastin time; *FBG* fibrinogen; *FDP* fibrin degradation product; *DD* D-dimer; *ATIII* antithrombin III; *ABE* actual base excess; *SBE* standard base excess; *Lac* lactate

**Fig. 1:**
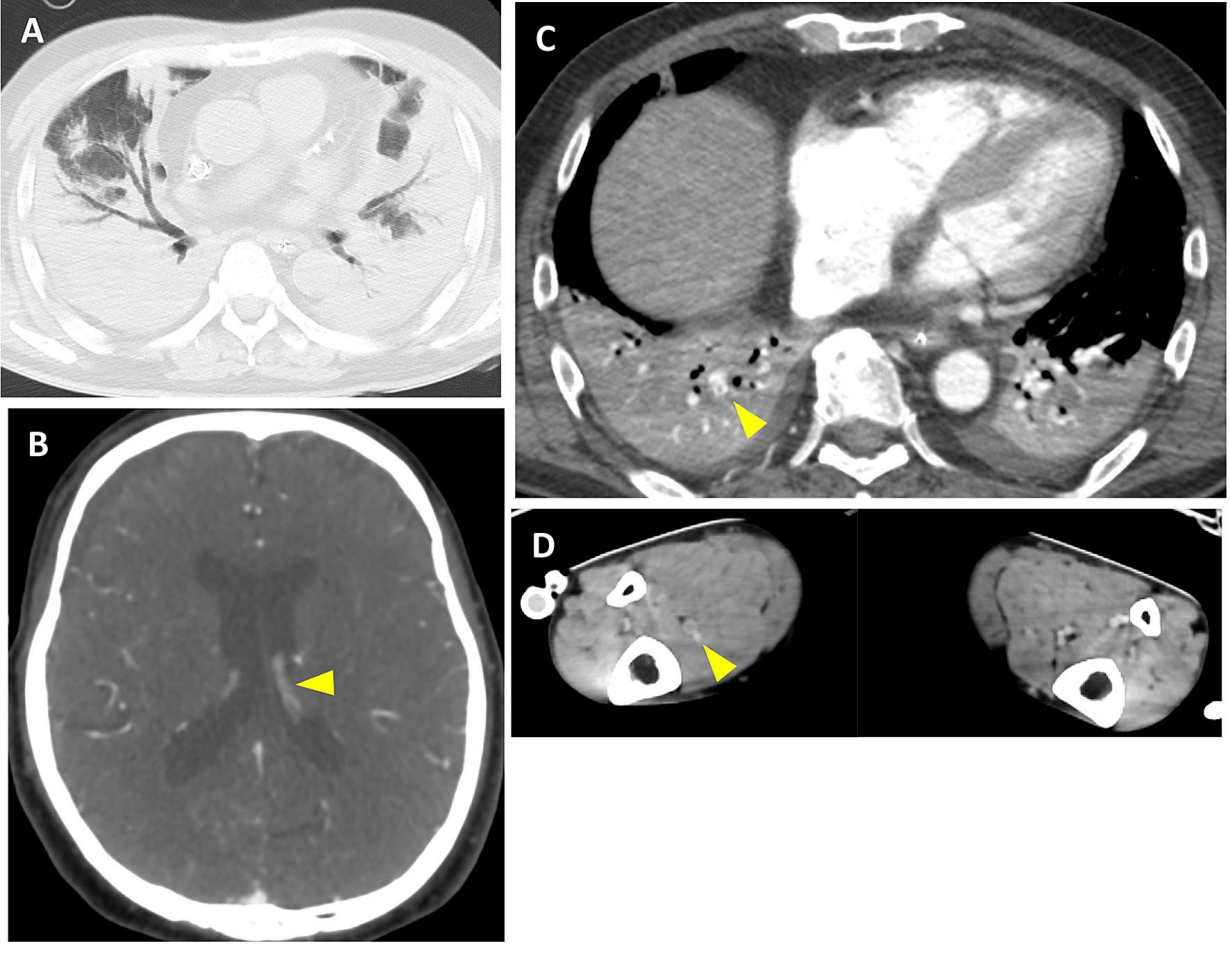
Computed tomography (CT) images. **a** CT scan showing bilateral and peripheral ground-glass and consolidative pulmonary opacities. CT scan showing **b** subarachnoid hemorrhage, multiple thrombosis in the **c** peripheral branch of the right pulmonary artery and **d** right internal jugular vein and right soleus vein

A computed tomography (CT) scan revealed bilateral and peripheral ground-glass and consolidative pulmonary opacities (Fig. 1a), subarachnoid hemorrhage, and multiple thromboses in the peripheral branch of the right pulmonary artery, right internal jugular vein, and right soleus vein (Fig. 1b).

During his management with ECMO, melena was observed, and the patient remained anemic (hemoglobin of 10.1 g/dL) despite repeated blood transfusions.

Therefore, the intensive care unit doctors consulted gastroenterologists to identify the source of gastrointestinal bleeding. On the sixth day of hospitalization, a semi-urgent esophagogastroduodenoscopy (EGD) was performed due to the patient’s progressive anemia.

To ensure personnel’s safety the endoscopy was performed using personal protective equipment (PPE), including the protective medical standard mask (N95), disposable surgical cap, face shield, goggles, disposable long sleeve water-resistant gown, and two pairs of gloves as recommended by the Japanese Gastroenterological Endoscopy Society.

**Fig. 2:**
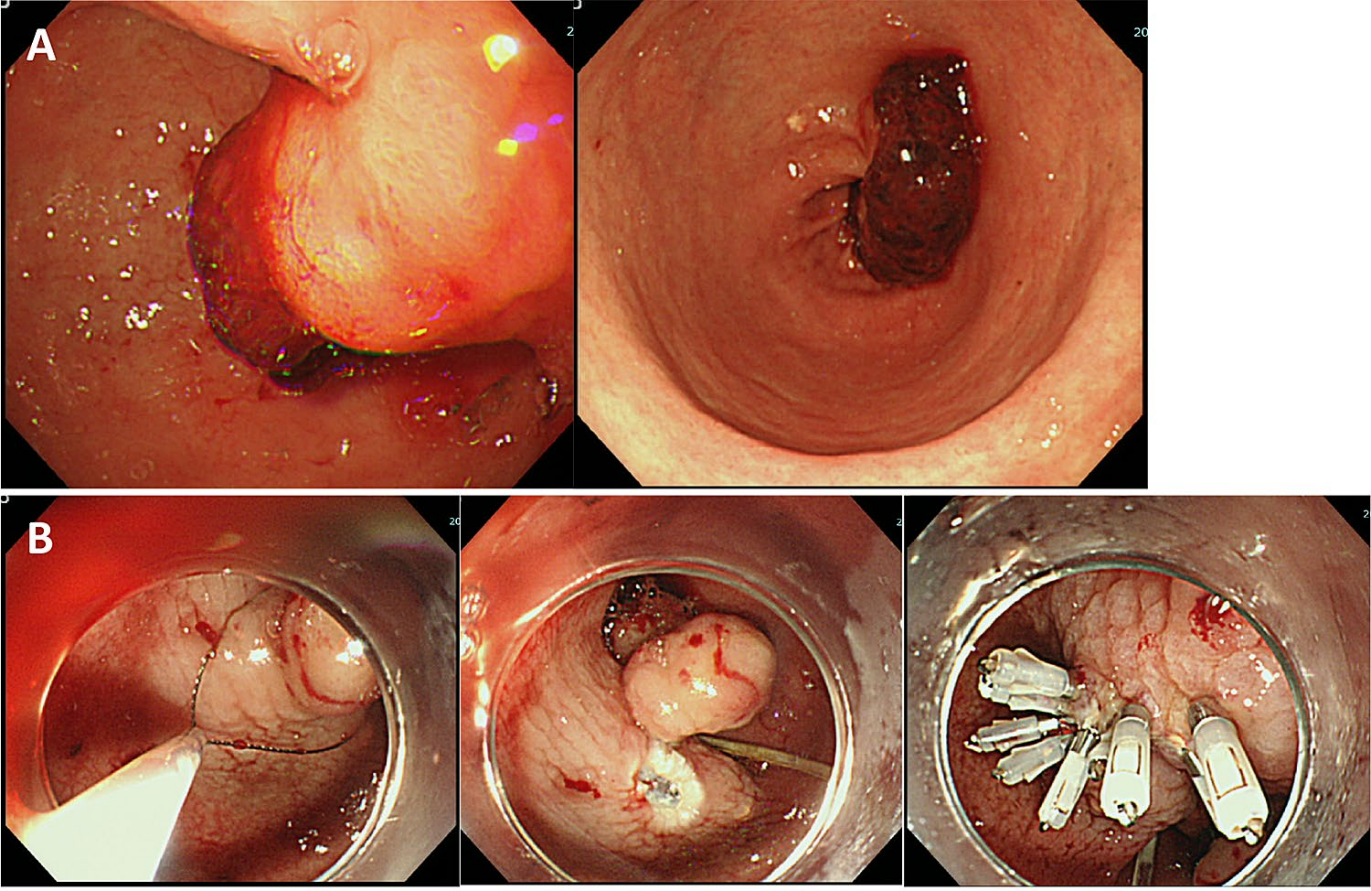
Esophagogastroduodenoscopic (EGD) findings. **a** EGD showing a pedunculated polyp with a diameter of 45 mm in the antrum of the stomach and a large blood clot on the surface. **b** Performing the endoscopic resection for bleeding gastric polyp for hemostasis

EGD revealed a pedunculated polyp with a diameter of 45 mm in the antrum of the stomach, with a large blood clot on the polyp surface, indicating that the patient’s progressive anemia was caused by hemorrhage from this polyp. A polypectomy performed and achieved hemostasis uneventfully (Fig. 2).

**Fig. 3:**
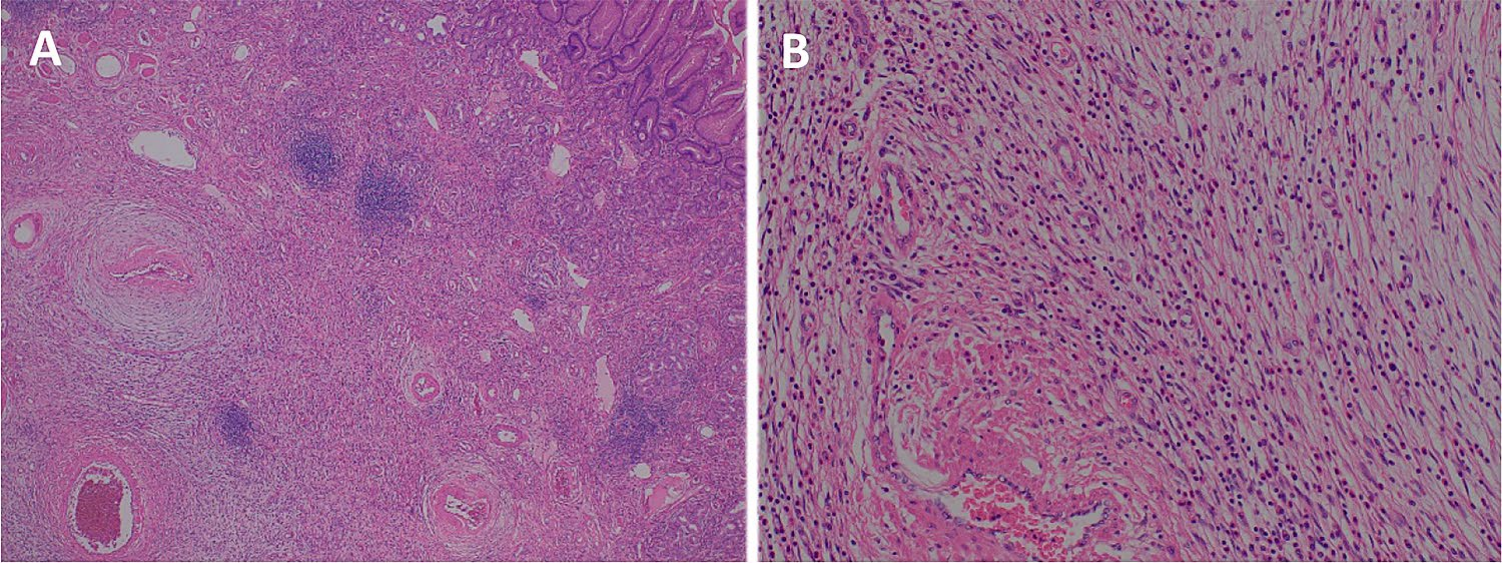
Histological findings of endoscopically resected specimen of the gastric polyp. **a** Low magnification, submucosal tumor with bland spindle sells, collagenous stroma were observed. **b** High magnification, fibrous tissue and infiltration of eosinophils were observed

**Fig. 4:**
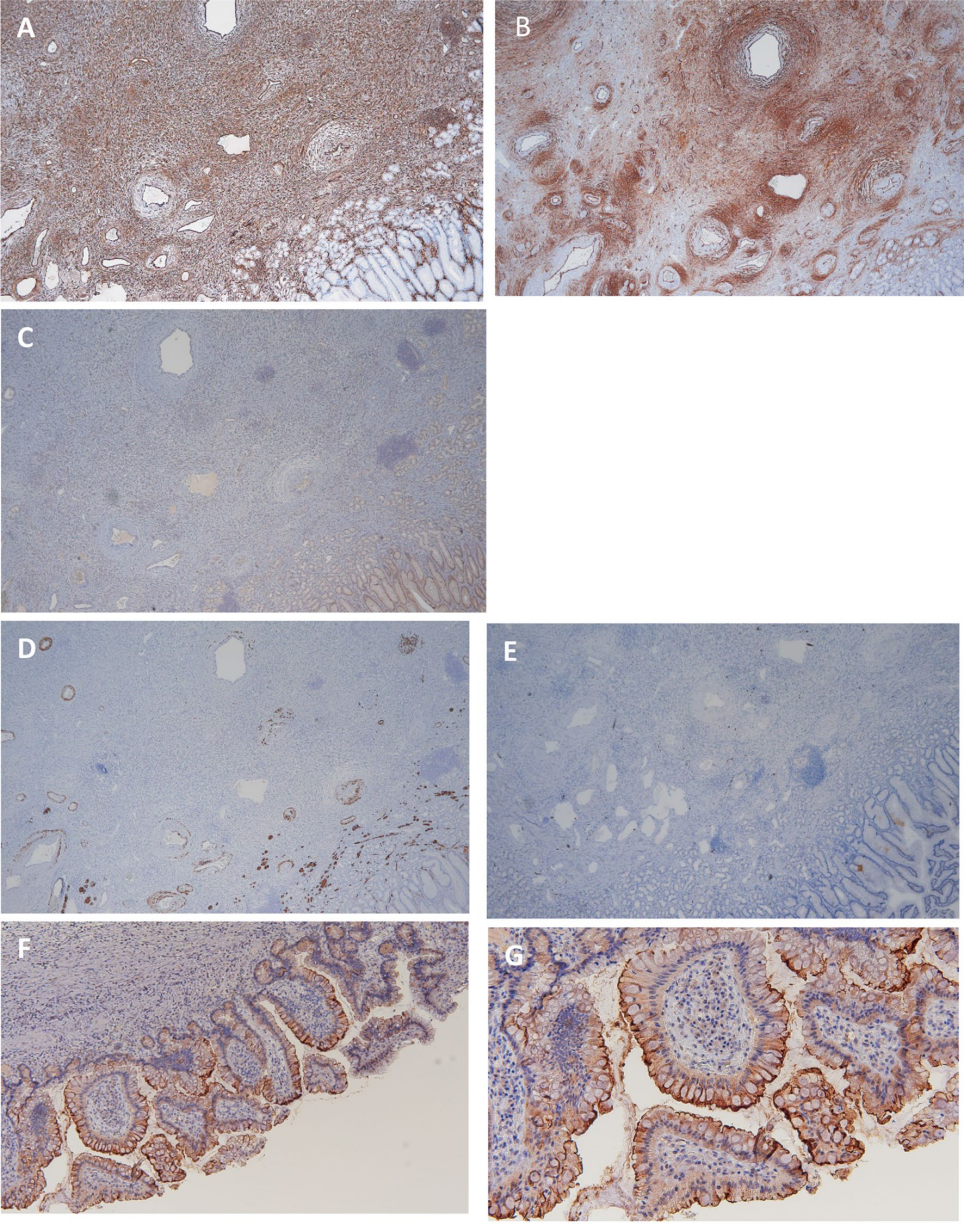
Immunohistochemical staining. Immunohistochemical staining showed that **a** CD34 and **b** vimentin were positive, but **c** DOG1, **d** S100, and **e** desmin were negative for the spindle cells. ACE2 was positive for epithelial cells (**f** low magnification, **g** high magnification)

Histological findings revealed a submucosal tumor containing fibroblast-like spindle cells with infiltration of eosinophils (Fig. 3). Immunohistochemical examination revealed that the spindle cells were positive for CD34 and vimentin, but negative for DOG1, S100, and desmin. The Ki-67 labeling index in the spindle cells was approximately 5%. Histological diagnosis was an inflammatory fibroid polyp (IFP). (Fig. 4).

## Discussion

We present the first report regarding endoscopic intervention for a COVID-19 patient with GI bleeding under ECMO treatment. A semi-emergent EGD revealed the cause of anemia was bleeding from a gastric IFP and a polypectomy was successfully performed resulting in endoscopic hemostasis. Importantly, we were able to safely perform endoscopic intervention with appropriate arrangements, even for a patient with COVID-19.

The COVID-19 pandemic has caused an unprecedented human and health crisis. This pandemic immediately affected all physicians, including gastroenterologists, worldwide since COVID-19 can be associated with triggering inflammation in the respiratory and GI systems. The reason SARS-CoV-2 infection affects the GI tract is due to the broad expression of angiotensin-converting enzyme 2 (ACE2) in the intestine ACE2 plays a role as an entry receptor for SARS-CoV-2 [3, 4]. GI symptoms in COVID-19 patients are hypothesized to result from intestinal damage due to a combination of the aggressive immune response against SARS-CoV-2, direct viral invasion, and tissue hypoxia caused by long-term hypoxemia related to severe pneumonia. Based on this mechanism, it is conceivable that GI bleeding, such as hematemesis and hematochezia, can occur in COVID-19 patients. There have been several reports regarding GI complications in patients with COVID-19, notably a recent report by Tian et al. [5] indicated that GI bleeding was present in 4–13.7% of COVID-19 patients and that GI symptoms—including GI bleeding—were more frequent in patients with severe COVID-19 than those with non-severe COVID-19. An autopsy of a patient who died due to COVID-19 reported histological degeneration and necrosis with varying degrees of mucosal damage. In this case, EMR was effective for stopping progressive anemia. However, we need to carefully observe patients with severe COVID-19 like this because they might accompany mucosal injury of all GI sites.

Although the initial coagulopathy of COVID-19 usually presents with a prominent elevation of D-dimer and fibrin/fibrinogen-degradation products [6], these were not present in our patient. Rather, we found bleeding from gastric IFP to be the cause of the patient’s progressive anemia. Massive hemorrhage from IFP is rare[7], therefore, we hypothesize it is due to a combination of the following factors: (1) direct GI mucosal damage due to viral invasion; (2) the strangulation of this IFP in conditions such as ball valve syndrome; (3) GI tissue hypoxia caused by long-term hypoxemia from respiratory failure; (4) COVID-19 induced coagulopathy in patients; and (5) use of steroids for prophylaxis of disseminated intravascular thrombosis.

In this case, we could not detect SARS-Cov2 in the epithelial cells of IFP and gastoric mucosa by in situ hybridization with RNAscope® Target Probe-V-nCoV2019-S (Advanced Cell Diagnostics, a brand of Bio-Techne Corporation, Newark, CA, USA). However, the present result does not rule out SARS-Cov2-related mucosal GI damage of bleeding from IFP because the virus may have already been eliminated at the time of endoscopic treatment. Thus, this case has a complicated etiology of GI bleeding from IFP.

Since the outbreak of COVID-19, screening endoscopic examinations have been suspended. Only patients with GI bleeding are eligible for emergent endoscopy; however, considering the possibility that every patient with COVID-19 undergoing an endoscopy can infect others, care must be taken when performing endoscopic examinations. In this case, we performed endoscopic examination wearing PPE in accordance with the Japanese Gastroenterological Endoscopy Society guidelines, and We judged that all staffs did not have COVID-19 because no one had any symptoms related to COVID-19 such as fever, respiratory, and gastrointestinal symptoms.

This case report highlights the necessity of endoscopic examination and treatment in COVID-19 patients, and how following strict guidelines can ensure that these procedures do not infect the staff involved in them. To afford our patients the best outcomes, we must identify endoscopic characteristics and treatments in COVID-19 patients with GI involvement.

## Abbreviations

ACE2: Angiotensin-converting enzyme 2
COVID-19: Coronavirus disease-2019
EGD: Esophagogastroduodenoscopy
ECMO: Extracorporeal membrane oxygenation
GI: Gastrointestinal
IFP: Inflammatory fibroid polyp
PPE: Personal protective equipment
SARS-CoV-2: Severe acute respiratory syndrome coronavirus 2
VV-ECMO: Venovenous extracorporeal membrane oxygenation
